# SolPredictor: Predicting Solubility with Residual Gated Graph Neural Network

**DOI:** 10.3390/ijms25020715

**Published:** 2024-01-05

**Authors:** Waqar Ahmad, Hilal Tayara, HyunJoo Shim, Kil To Chong

**Affiliations:** 1 Department of Electronics and Information Engineering, Jeonbuk National University, Jeonju 54896, Republic of Korea; 2 School of International Engineering and Science, Jeonbuk National University, Jeonju 54896, Republic of Korea; 3 School of Pharmacy, Jeonbuk National University, Jeonju 54896, Republic of Korea; 4 Advanced Electronics and Information Research Center, Jeonbuk National University, Jeonju 54896, Republic of Korea

**Keywords:** molecular solubility, drug discovery, ADMET, artificial intelligence, regression, graph neural network, residual gated graph neural network, simplified molecular-input line-entry system

## Abstract

Computational methods play a pivotal role in the pursuit of efficient drug discovery, enabling the rapid assessment of compound properties before costly and time-consuming laboratory experiments. With the advent of technology and large data availability, machine and deep learning methods have proven efficient in predicting molecular solubility. High-precision in silico solubility prediction has revolutionized drug development by enhancing formulation design, guiding lead optimization, and predicting pharmacokinetic parameters. These benefits result in considerable cost and time savings, resulting in a more efficient and shortened drug development process. The proposed SolPredictor is designed with the aim of developing a computational model for solubility prediction. The model is based on residual graph neural network convolution (RGNN). The RGNNs were designed to capture long-range dependencies in graph-structured data. Residual connections enable information to be utilized over various layers, allowing the model to capture and preserve essential features and patterns scattered throughout the network. The two largest datasets available to date are compiled, and the model uses a simplified molecular-input line-entry system (SMILES) representation. SolPredictor uses the ten-fold split cross-validation Pearson correlation coefficient $R^{2}$ 0.79±0.02 and root mean square error (RMSE) 1.03±0.04. The proposed model was evaluated using five independent datasets. Error analysis, hyperparameter optimization analysis, and model explainability were used to determine the molecular features that were most valuable for prediction.

## 1. Introduction

Drug discovery is the process of identifying and developing new drugs for the treatment and prevention of diseases. This process has a significant impact on human health and society. Drug discovery drives innovation, meets medical needs, positively affects the economy, and improves public health.

Solubility is an important parameter in drug discovery for several reasons. Solubility affects the bioavailability, synthesis, and manufacture of drugs as well as the different drug design stages [1]. Chemists seek to improve the solubility of molecules through molecular structure optimization during drug design [2]. When a drug-like compound has adequate ADMET (absorption, distribution, metabolism, excretion, and toxicity) properties, the compound can be developed as a new medication [3]. Solubility is a major factor influencing drug absorption [4]. The solubility of a drug in intestinal fluid [5] is a prerequisite for achieving both concentration in the blood and therapeutic effects. Understanding the solubility of a compound enables chemical teams to make appropriate decisions during the early stages of drug development. Poorly soluble compounds may be discarded in the early stages of drug development in order to save time and cost. Solubility is a very important physicochemical parameter and driver in drug bioavailability, and is considered during the early-stage drug screening process [6]. Although traditional methods of determining solubility are time consuming and costly [7], with recent technological advancements it is now possible to develop computational methods to minimize the time and cost of traditional methods, and in certain cases replace experimental work. Computational methods are data-driven, and are trained using physicochemical properties and molecular representations. These computational methods are trained for physicochemical properties and molecule representation. Over the past two decades, owing to technological advancements in every field of life, a large amount of data has been produced. Traditional analytical techniques are inadequate for processing this large amount of data [8]. Several machine learning (ML) approaches have been used to predict the molecular properties of materials, including their toxicity, solubility, lipophilicity, bandgaps, and conductivity, as well as protein structures [9,10,11,12,13]. ML models use different molecular representations and designs, including fingerprint representation with artificial neural network (ANN), a combination of multiple linear regression (MLP), ANN, and complex deep networks [14,15,16,17], and molecular descriptors of physicochemical properties, fingerprints, and topological indices. SMILES representations use recurrent neural networks (RNN), long short-memory models (LSTM), and the gated current unit (GRU) [18,19,20].

Graph-based representations explicitly represent a molecule’s structural characteristics such as complex ring structures, branching, and other spatial connections between atoms. A neural network that is designed specifically to handle graph-structured data, with atoms representing nodes and edges expressing connections among them, is called a graph neural network (GNN). GNNs can learn spatial patterns and relationships among atoms. Different GNN architectures have been studied and utilized for solubility prediction. Graphically represented models use graph convolutional networks (GCNs), graph attention networks (GATs), and graph transformers [21,22,23]. Considerable research effort has been expended in developing computational models for solubility prediction [10,24,25,26]. Early solubility computational models utilized molecular, electronic, and structural properties as inputs [27,28], represented molecules as 2D/3D images [29], or used the simplified molecular-input line-entry system (SMILES), SELF-referencing embedded strings (SELFIES), or SMILES arbitrary target specifications (SMARTS) [30,31,32]. For solubility determination, Gao et al. [33] proposed a deep neural network for molecular property prediction using m-ordered descriptors and a graph convolution network using graphs. The effectiveness of both deep neural networks (DNNs) and GCNs for solubility prediction was then compared. Cui et al. [34] applied a deeper-net model to the 9943 training set. Bae et al. [35] developed support vector machine (SVM), gradient boosting tree (GBT), k-nearest neighbors (kNN), multilayer perceptron (MLP), and random forest (RF) models based on five fingerprint variants (RDKit, Morgan, Layered, Pattern, and MACCS). Maziarka et al. [36] developed a molecular attention transformer (MAT) architecture based on the present state-of-the-art transformer architecture for natural language processing services. MAT applies self-attention to the molecular graph representation of a molecule, while a feature vector identifies each node. This feature vector is then paired with a matrix called the adjacency matrix, which represents the molecule’s relationship, and a distance matrix that represents the distance between each atom in the resulting 3D conformer of the molecule. SolTranNet [37] is a machine learning network based on the MAT architecture that estimates the solubility of a material in aqueous systems and is trained using AqSolDB. The RMSE and $R^{2}$ of SolTransNet were 1.459 and 0.6764, respectively, using cross-validation on the AqSolDB dataset.

We combined the Cui et al. [34] and AqSolDB [38] datasets and propose a residual-gated graph neural network (RGNN). The RGNN obtains both temporal and structural dependencies. Five independent datasets (Cui 2020 [34], Boobier 2017 [39], Lovric 2020 [40], Llinas 2020 set1, and set2 [41]) were evaluated, with $R^{2}$ values of 0.547, 0.814, 0.805, 0.373, and 0.677 and RMSE values of 0.597, 0.743, 0.783, 0.991, and 1.142, respectively. Overall, molecular solubility is a critical parameter in drug discovery that influences bioavailability, formulation development, synthesis, manufacture, structure–activity relationship (SAR) studies, ADMET profiling, and lead optimization. By addressing solubility challenges and optimizing the solubility characteristics of drug candidates, researchers can increase the likelihood of successful drug development and improve the therapeutic potential of new medications.

## 2. Results and Discussion

SolPredictor was evaluated based on two metrics: $R^{2}$ and RMSE. The correlational measures allow us to determine how effectively the models rank the compounds in terms of solubility, whereas the error metric allows us to assess the average degree of error in the model predictions. Table 1 shows the ten-fold RMSE and $R^{2}$. The RMSE values ranged from 0.93 to 1.09, with an average of 1.03±0.04. Fold 2 had the minimum RMSE, whereas fold 10 had the maximum RMSE value. The $R^{2}$ values ranged from 0.73 to 0.81, with an average $R^{2}$0.79±0.02. Fold 5 had the maximum $R^{2}$ value, whereas fold 1 had the minimum value of 0.73.

**Figure 1:**
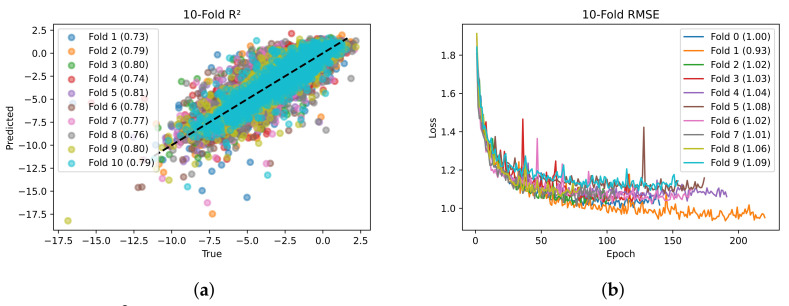
(**a**) $R^{2}$ scatter plot and (**b**) validation loss from ten-fold cross-validation.

The scatter plot in Figure 1 shows the relationship between the actual labels and model prediction values. The $R^{2}$ values are shown for each fold. There is a strong pattern in which the predicted values increase as the actual data values increase, depicting a strong relationship. Furthermore, the line with a positive slope from the bottom left to the top right of the plot’s points shows a positive correlation. The datapoints are tightly concentrated around the line of best fit, demonstrating the strength of the relationship. When two variables on a scatter plot have a significant positive correlation, it means that the relationship between them is consistent and tends to increase as one variable rises.

### 2.1. Evaluation of Independent Datasets

The results for the independent dataset are summarized in Table 2. The $R^{2}$ and RMSE results for SolPredictor were compared with SolTranNet. The number of molecules used in the present model prediction may differ from those used by other authors owing to cleaning and duplication removal. Each row in Table 2 shows the best $R^{2}$ and RMSE values in bold.

### 2.2. Error Analysis

**Figure 2:**
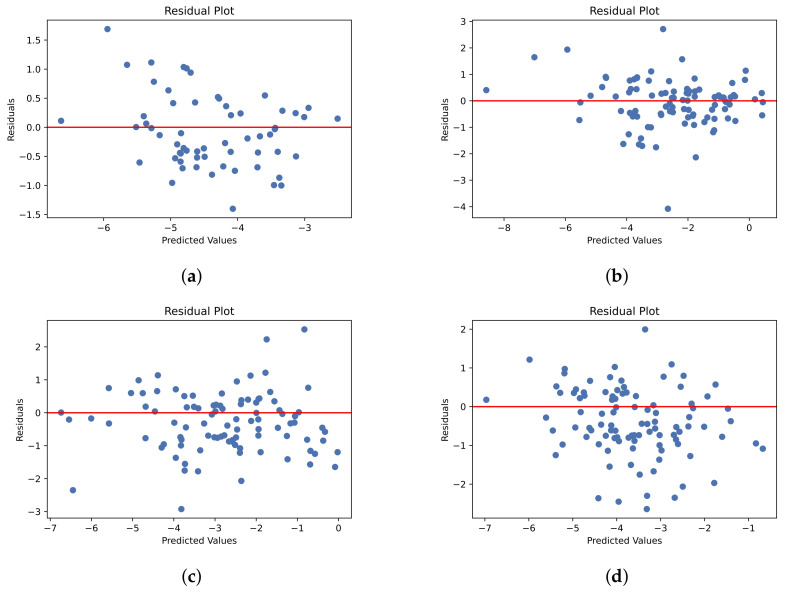
Residual plots for test sets: (**a**) Cui et al.; (**b**) Lovric; (**c**) Boobier; (**d**) Llinas.

Scatter charts were constructed for the test set residuals and predicted values. Residuals are the differences between the actual values (labels) and model predictions. Residual plots are used to visually observe the regression model errors. The plots show the model predictions as independent variables and the residuals as the dependent variable. The residual values are randomly distributed around the horizontal zero line. If the residuals have a U-shaped pattern, then the model does not capture nonlinear relationships between dependent and independent variables, whereas a funnel-shaped pattern shows that either the use of another model or data transformation is required. Figure 2 shows that the values are distributed randomly around the horizontal line, meaning that the model displayed a random pattern with no evident systematic structure.

### 2.3. Feature Importance

**Figure 3:**
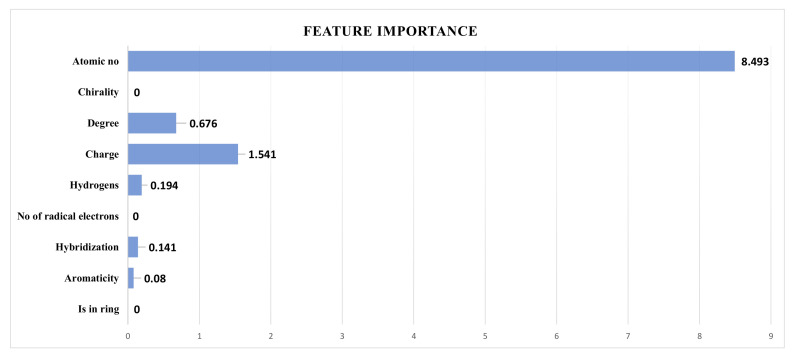
Feature importance chart, showing that atomic number is the most important node feature.

GNNs have become popular for processing graph-structured data. Because of the complexity of graph-based data and the nonlinear interactions between graph nodes, it might be difficult to explain why a GNN generates a specific prediction. Hence, there are explanatory problems for GNNs, and it is difficult to explain how these models make predictions. GraphFramX [42] provides explainability techniques. Traditionally there are two ways to explain GNNs, local explanation and second global explanation. Local explanation emphasizes a single node or edge, whereas the global approach uses the overall GNN behavior. Herein, local phenomena were used for SolPredictor explanation. We used the Pyg captum explainability module to explain the feature importance. The model configurations were regression, graph, and raw for the mode, task level, and return type, respectively. SolPredictor uses nine types of node features. As shown in the Figure 3, the explainability mechanism shows that the atomic number is the most important node property.

### 2.4. Hyperparameter Tuning

**Figure 4:**
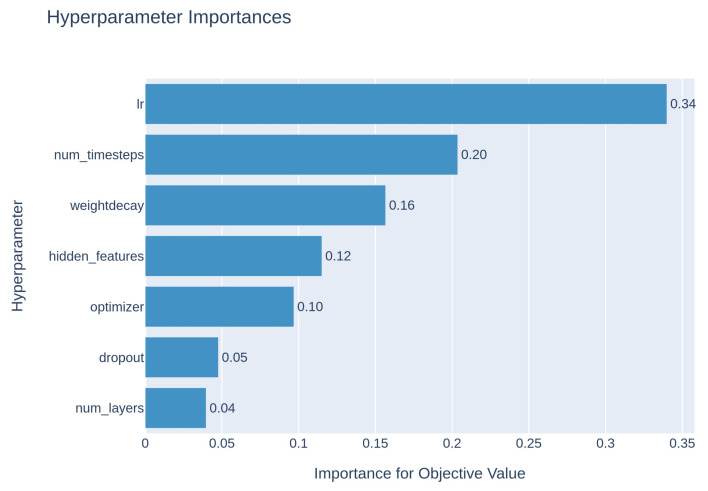
Importance of hyperparameters, showing that the learning rate is most important.

The best model architecture for a specific model is not always clear. Therefore, we investigated several choices. In a typical machine learning method, the machine is tasked to help with this exploration and to dynamically select the optimal model architecture. The hyperparameters determine the model architecture, and the process of obtaining the best model architecture is known as hyperparameter tuning. The hyperparameters were tuned for the SolPredictor using Optuna [43]. Optuna is a software (v.3.5.0) framework that facilitates automatic hyperparameter adaptability. The hyperparameters for SolPredictor are the learning rate, weight decay, optimizer, hidden features, number of layers, number of time steps, and dropout. The hyperparameter ranges are presented in the Supplementary Materials. As shown in Figure 4, the learning rate is the most important hyperparameter, whereas the number of layers has the least effect on the model.

### 2.5. Web Server for Solubility

**Figure 5:**
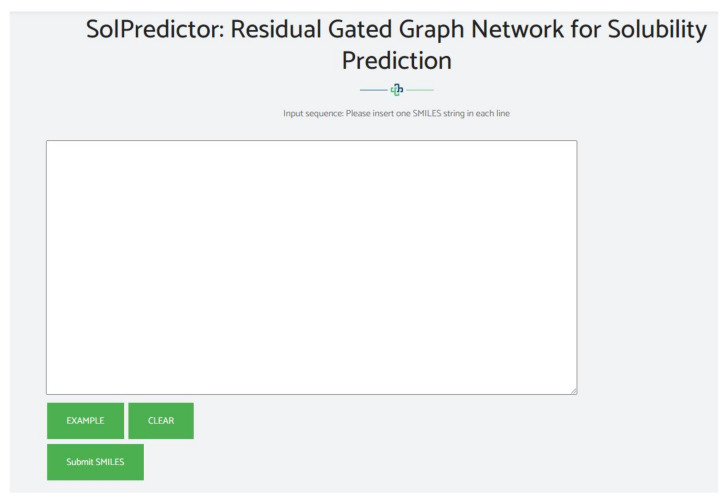
Web server for SolPredictor.

Finally, a web server was developed for researchers and pharmaceutical industry experts. The server was based on a residual graph convolution model. The page takes SMILES list input, then calculates and shows predictions. The web server is demonstrated in Figure 5. The web server tool is available at https://nsclbio.jbnu.ac.kr/tools/SolPredictor/ (accessed on: 2 January 2024).

## 3. Materials and Methods

### 3.1. Dataset

**Figure 6:**
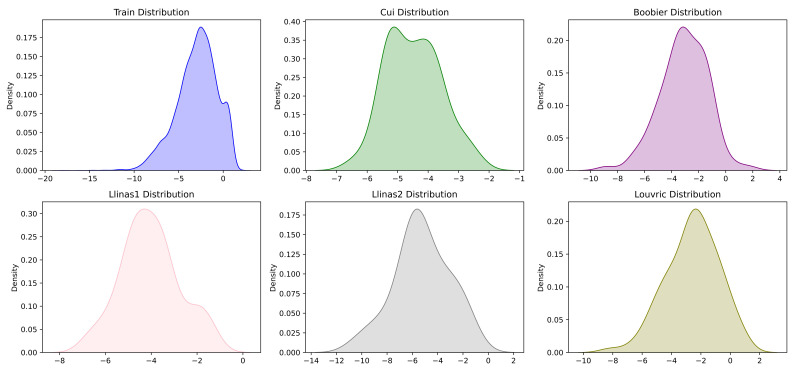
Solubility histograms: training dataset values ranged from −18.21 to 1.7 and test dataset values ranged from −6.52 to −2.36.

**Figure 7:**
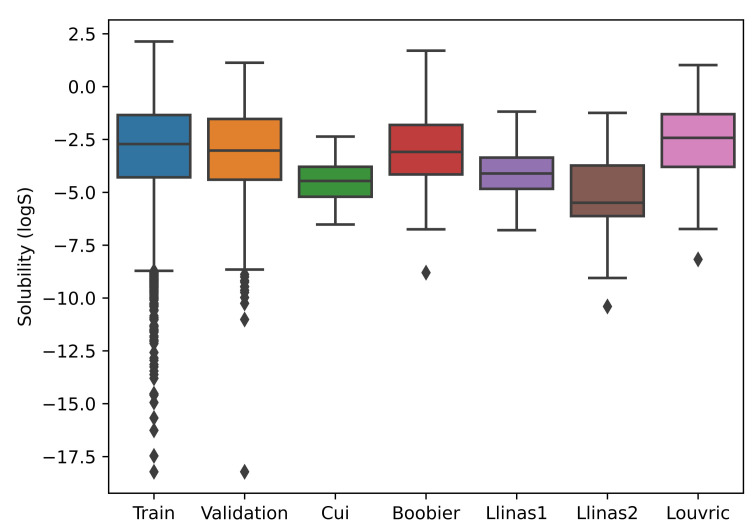
Boxplots for training, validation, and five test datasets. The training dataset had a median at −2.8, maximum at 2.3, and minimum at −8, with outliers at −9 to −17.5.

We learned that neural network models perform well with more datapoints; thus, we utilized the datasets of Cui et al. [34], AqSolDB [38], and Lovric [40] for SolPredictor training. The datasets contained 9943, 9982, and 583 datapoints, respectively. All datasets were concatenated and duplicate SMILES were removed. The final combined dataset contained 17,826 datapoints. The dataset was divided into 90% for training and 10% for validation. The validation dataset was used in the SolPredictor hyperparameter optimization process. Each record had a SMILES string and logS value. logS refers to the logarithm of the water solubility of a compound and is measured in mol/L; a higher logS indicates higher solubility and vice versa. The logS values ranged from −17.46 to 2.13. The length max, min, mean, std, and var for SMILES were 783, 2, 34, 25, and 662, respectively. The model was tested on five independent datasets from: Cui et al. [34], Boobier et al. [39], Lovric et al. [40], and Llinas et al. [41] set1 and set2, which contained 62, 95, 95, 99, and 32 datapoints, respectively. Each test dataset was checked against a training dataset for SMILES duplicates, and datapoints were deleted from the test dataset if any duplication was found. As shown in Figure 6, kernel density estimation plots represent the training, Cui2020 [34], Boobier2017 [39], Lovric2020 [40], and Llinas2020 set1 and set2 [41] test datasets. The *x*-axis represents the logS range, whereas the *y*-axis represents the density. The higher the *y*-axis value at a particular point on the *x*-axis, the higher the probability of that point. Figure 7 shows boxplots for the training and test datasets. Several of the logS values from the datasets of Cui et al. [34] and Llinas et al. [41] set2 datasets were non-overlapping with the training dataset. The Llinas et al. [41] set2 boxplot median line was lower in the interquartile range, which shows that the logS values are positively skewed.

#### 3.1.1. Data Preprocessing

**Figure 8:**
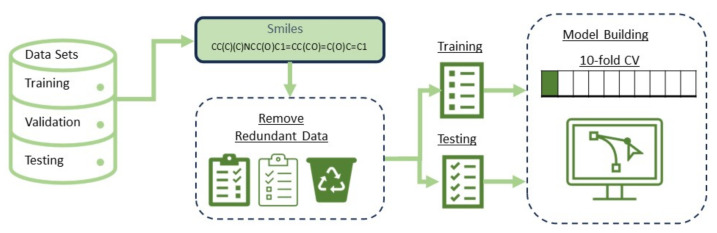
Data preprocessing flow chart showing molecule and graph generation from SMILES data.

In this step, the datasets of Cui et al., AqsolDB, and Lovric were combined and SMILES duplication was removed using Pandas [44]. SMILES entries with a length of 1 were removed as well, as a single-node graph was not valid. In implementing the model, the SMILES were input features and the logS values were labels. The model used a graph as the input. A graph is a data structure created using two-component nodes and edges. In a molecular context, nodes are atoms, whereas edges are bonds. The SMILES data were converted into molecules using the rdkit library [45] and checked for validity, then a graph object was generated using the node features in Table 3 and saved on disk. The SMILES data were converted to a graph using Pytorch Geometric (PyG) [46]. The SMILES data were processed one by one, as shown in Figure 8. An unambiguous graphical representation was obtained by assigning a number to each atom in the molecule and then traversing the molecular graph in that order.

#### 3.1.2. Ten-Fold Data Split

Cross-validation is a resampling method, commonly known as out-of-sample estimation or rotation estimation, which is used to evaluate how statistically-determined results generalize to independent datasets by testing machine learning models on a limited set of data. The algorithm has a single parameter k that determines the number of groups into which a given data sample must be divided. Therefore, this technique is commonly referred to as k-fold cross-validation. The entire training dataset was randomly divided into ten folds of equal size and independence, with no rows repeated in any other fold. The model was trained with nine folds and then tested with the remaining fold to obtain a performance score. This process was repeated ten times to create ten models and scores for each model.

### 3.2. Methods

Deep learning approaches have been shown to be effective in predicting the molecular properties of compounds [47], and are becoming a growing instrument in modern computer-aided drug design toolboxes. Because molecules can be represented as graphs, one apparent strategy for deep learning is to utilize a graph-based architecture, which enables the use of graph-based neural networks (GNNs).

#### 3.2.1. Molecular Feature Extraction

The extraction of molecular features is a vital step in the preparation of molecular data for graph neural network (GNN) models. Molecular atoms are the basic building blocks of SolPredictor. Nine types of atomic features were extracted; these are listed in Table 3. The feature vector encapsulates the features of each atom in a molecule. The atomic number, degree, formal charge, number of radical electrons, hybridization, aromaticity, hydrogen atoms, chirality, and ‘is in ring’ property are all common atomic-level characteristics. The ranges for the atomic number, degree, and charge are 1–119, 0–11, and −5 to 7, respectively. The aromaticity and ‘is in ring’ property have true or false values. The SMILES string was converted to the rdkit [45] molecule and the validity of the molecule was checked. The graph was created using the Pytorch geometric library. The atomic features were treated as node features in the graph; for example, if a molecule has 32 atoms, then the graph size is 32 × 9.

#### 3.2.2. Graph Neural Network (GNN)

A graph neural network (GNN) is a type of neural network specifically designed to operate on graph-structured data. GNNs obtain local and global interactions between atoms and their neighbors. The graphs consist of vertices (or nodes) and links (or edges) that represent the relationships between the vertices. GNNs are capable of learning and extracting meaningful representations from the nodes and edges of a graph, enabling them to perform various tasks such as graph regression/classification, node classification, link prediction, and graph generation.

(1) G=(V,E)

The graph consists of a set of nodes *V* and some edges *E* that connect them. Each node *V* has a predefined vector associated with it that encodes its state, that is, the node’s hidden state. Hidden state vectors are initialized with node-related features from the input graph at the start of GNN execution. Edges may include a set of features if desired. Following initialization of the hidden states, a message-passing algorithm is executed based on the connections of the input graph. The message-passing process comprises three major phases: (i) message, (ii) aggregation, and (iii) update. During the GNN message-passing phase, each vertex is updated according to the aggregation of the neighboring vertices. The embedding $h_{u}^{\left(k\right)}$ associated with vertex u∈V.

(2) $$\;\;\;h_{u}^{\left(l+1\right)}=update^{\left(l\right)}\left(h_{u}^{l},AGGREGATE^{\left(l\right)}\left(\left\{h_{v}^{l},\forall v\in N_{u}\right\}\right)\right)\;$$

(3) $$=update^{\left(l\right)}\left(h_{u}^{l},m_{N(u)}^{\left(l\right)}\right)$$

AGGREGATE and update are learnable functions and message m node u neighbors aggregation.

#### 3.2.3. Residual Gated Graph Convolution

An RNN is a neural network specifically designed for sequential or time-series data. Each datapoint input to the RNN depends on the previous data input. RNNs maintain hidden feature vectors or contexts from previous inputs. At each time step, the RNN takes the input vector and hidden feature vector and produces the output along with the updated hidden vector:

(4) $$h_{i}=f_{RNN}\left(w_{i},\left\{h_{j}:j=i-1\right\}\right)$$

where the vector $h_{i}$ for word i is computed from the previous feature vector $h_{j}$ and word $w_{i}$ in the sequence. Graph RNN, introduced in 2005 [48], proposed a multilayer perceptron with an RNN on a graph. In this case, the feature vector $h_{i}$ of vertex *i* is

(5) $$h_{i}=f_{GRNN}\left(w_{i},\left\{h_{j}:j\to i\right\}\right)=\sum_{j\to i}C_{GRNN}\left(w_{i},h_{j}\right),\;$$

where $w_{i}$ is a feature vector for the current vertex and $h_{j}$ is the feature vector set of the current vector neighbor’s feature vector vertices. Here, $C_{GRNN}\left(w_{i},h_{j}\right)$ is defined as

(6) $$C_{GRNN}\left(w_{i},h_{j}\right)=P\sigma \left(Q\sigma \left(Rx_{i}+Sh_{j}\right)\right),\;$$

where *P*, *Q*, *R*, and *S* are weighted parameters and σ is a sigmoid function. In 2016, Sukhbaatar et al. [49] proposed the ConvNet graph, represented as follows:

(7) $$h_{i}^{l+1}=f_{GCNN}\left(w_{i}^{l},\left\{h_{j}^{l}:j=i\to 1\right\}\right)=ReLU\left(Q^{l}h_{i}^{l}+R^{l}\sum_{j\to i}h_{j}^{l}\right),\;$$

where ReLU is the rectified linear unit and *l* represents the layer level. In 2017, Marcheggiani et al. [50] proposed a function for edge gate importance:

(8) $$h_{i}^{l+1}=f_{GCNN}\left(w_{i}^{l},\left\{h_{j}^{l}:j=i\to 1\right\}\right)=ReLU\left(\sum_{j\to i}\eta_{ij}h_{j}^{l}\right),\;$$

where $\eta_{ij}$ works as an edge gate and is represented as

(9) $$\eta_{ij}=\sigma \left(Ch_{i}^{l}+D^{l}h_{j}^{l}\right).\;$$

The gated graph convolution is

(10) $$h_{i}^{l+1}=f_{GGCNN}\left(w_{i}^{l},\left\{h_{j}^{l}:j=i\to 1\right\}\right)=ReLU\left(R^{l}h_{i}^{l}+\sum_{j\to i}\eta_{ij}h_{j}^{l}\right).\;$$

This equation has a source vertex, neighboring vertex information, and edge gate capability. Subsequently Bresson and Laurent [51] formulated a residual gated graph convolution by adding an identity operator between the layers:

(11) $$h_{i}^{l+1}=f^{l}\left(h_{i}^{l},\left\{h_{j}^{l}:j\to i\right\}\right)+h_{i}^{l}\;$$

where $h_{i}^{l+1}$ represents the node features for the l+1 layer, $h_{i}^{l}$ represents the node features for the current layer *l*, and $h_{j}^{l}$ represents the neighboring nodes. Multiple layers of the RGNN can be stacked iteratively using Equation (11) to capture and transfer information across the graph structure.

#### 3.2.4. Implementation Details

**Figure 9:**
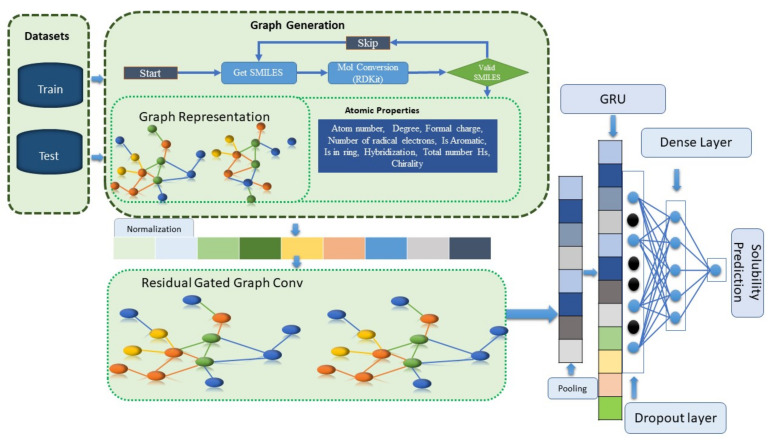
Flow diagram showing conversion of SMILES to graph and application of RGNN layers.

A model was developed for solubility prediction using a residual gated graph neural network. As shown in Figure 9, SMILES data were converted to graphs using the PyG library. The model uses nine node features as inputs. The features were normalized and passed to the linear layer, and the RGNN convolution was applied. After global pooling, embedding was employed as a sequence and the GRU was used to capture long-term dependencies. Finally, a dense layer was used to calculate the solubility.

#### 3.2.5. Evaluation Metrics

RMSE and $R^{2}$ were used as metrics to evaluate the performance of the SolPredictor regression model. RMSE is the square root of the average difference between the expected and actual values of a target variable. It is a statistical measure of the extent to which the predicted values of a model diverge from the actual values. The RMSE is always positive; it measures how close the predicted and true values are, with lower values indicating better model performance. The RMSE is calculated as follows:

(12) $$RMSE\;\;=\;\;\sqrt{\frac{\sum_{i=0}^{n}{\left(y_{i}-\hat{y_{i}}\right)}^{2}}{n}}\;$$

The Pearson correlation coefficient is frequently used in statistics, data analysis, and machine learning to assess the linear relationships between variables. $R^{2}$ indicates the correlation between two variables, namely, the model predictions and actual label values. $R^{2}$ ranges from −1 to +1, and indicates the direction and strength of the variables. The direction of this relationship is represented by a coefficient; a positive sign implies a positive connection, whereas a negative sign indicates a negative correlation.

(13) $$R^{2}\;=\;\;1-\frac{\sum_{i=0}^{n}{\left(y_{i}-\hat{y_{i}}\right)}^{2}}{\sum_{i=0}^{n}{\left(y_{i}-\bar{y}\right)}^{2}}$$

## 4. Conclusions

Aqueous solubility is crucial in physical chemistry. The in silico prediction of solubility with a high degree of reliability may significantly reduce the cost and time required for developing medications. In this paper, the SolPredictor model is proposed based on a RGNN. During the graph convolution process, the gating mechanism of the RGNN allows the model to emphasize local and global information selectively. This gating technique regulates the flow of data, allowing the model to focus on relevant network neighbors or to collect data from distant nodes. This feature enables SolPredictor to capture both local and global dependencies in a graph, which improves the ability of the model to grasp and describe complicated graph patterns and build deeper network robustness for noisy or incomplete data. Other tasks that could benefit from the improved molecular representation of AI models include yield prediction, synthesis planning, toxicity prediction, reaction outcome prediction, bioactivity prediction, and retrosynthesis analysis. SolPredictor demonstrated a ten-fold split cross-validation coefficient of $R^{2}$ 0.79±0.02 and root mean square error (RMSE) of 1.03±0.04. The model was tested on five independent datasets: Cui et al. [34], Boobier et al. [39], Lovric et al. [40], and set 1 and set2 from Llinas et al. [41]. Additional chemical representation features (e.g., bond features) or additional experimental solubility data may improve the solubility prediction ability of this model. The method shown here can be extended to the development of other high performance deep learning models for physical chemistry problems, such as toxicity and lipophilicity prediction. 

## Figures and Tables

**Table 1:** SolPredictor ten-fold cross-validation performance; fold 2 has the lowest RMSE.

Fold	1	2	3	4	5	6	7	8	9	10
RMSE	1.00	0.93	1.02	1.03	1.04	1.08	1.02	1.01	1.06	1.09
$R^{2}$	0.73	0.79	0.80	0.74	0.81	0.78	0.77	0.76	0.80	0.79

**Table 2:** Comparison of $R^{2}$ and RMSE across independent datasets.

Datasets	SolTranNet		SolPredictor	
	$R^{2}$	**RMSE**	$R^{2}$	**RMSE**
Cui et al. [34]	**0.611**	0.624	0.547	**0.597**
Boobier et al. [39]	0.724	1.010	**0.814**	**0.743**
Lovric et al. [40]	0.783	**0.720**	**0.805**	0.783
Llinas et al. [41] set1	**0.527**	**0.952**	0.373	0.991
Llinas et al. [41] set2	**0.824**	1.243	0.677	**1.142**

**Table 3:** Nine features of atoms.

Atom Features	Description
Atom number	1 to 119
Chirality	Atom chirality
Degree	0 to 11
Charge	−5 to 7
Hydrogens	Connected hydrogens
No of radical electrons	0 to 5
Hybridization	s, sp2, sp3d2, sp, sp3, sp3d, other
Aromaticity	False or True
Is in ring	False or True

## Data Availability

Code and datasets are available at SolPredictor (https://github.com/waqarahmadm019/SolPredictor, accessed on 2 January 2024).

## Supplementary Materials

The following supporting information can be downloaded at: https://www.mdpi.com/article/10.3390/ijms25020715/s1.
