# Supplementary material for: SolPredictor: Predicting Solubility with Residual Gated Graph Neural Network
Source: Int J Mol Sci. 2024 Jan 5;25(2):715. doi: 10.3390/ijms25020715 (PMC10815788; doi:10.3390/ijms25020715)
Supplement: Supplementary file 1 [file ijms-25-00715-s001.zip › ijms-2785401-supplementary.pdf]

Supporting information: Optuna visualizations.

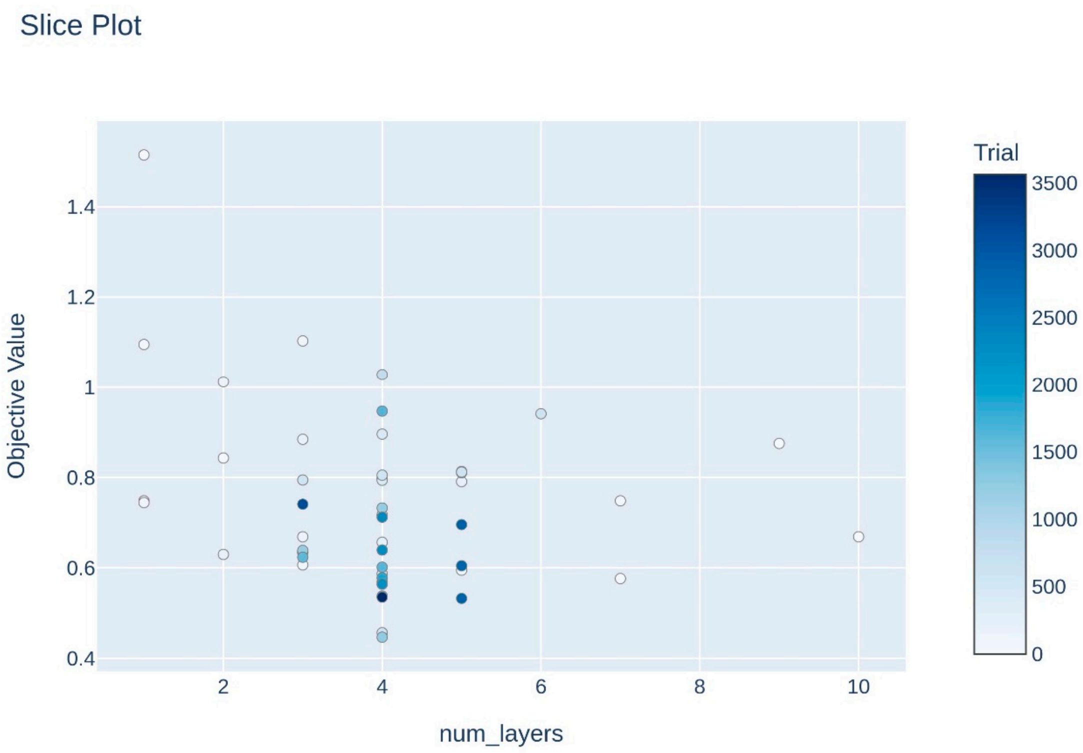

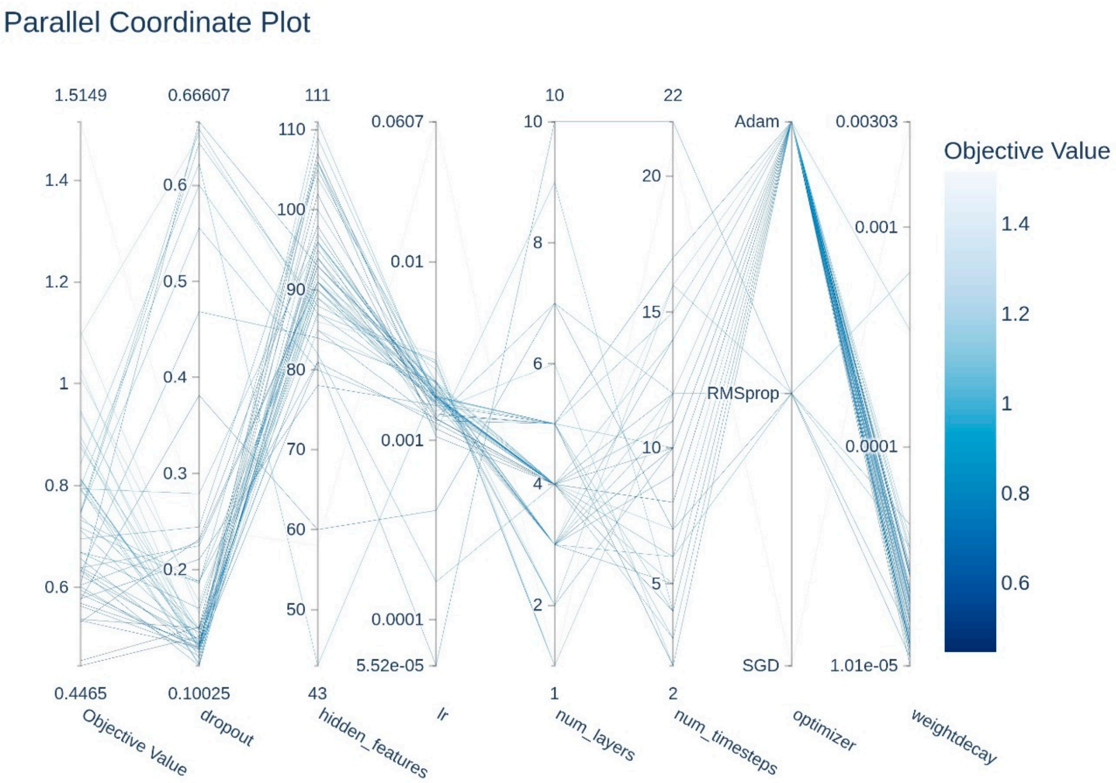

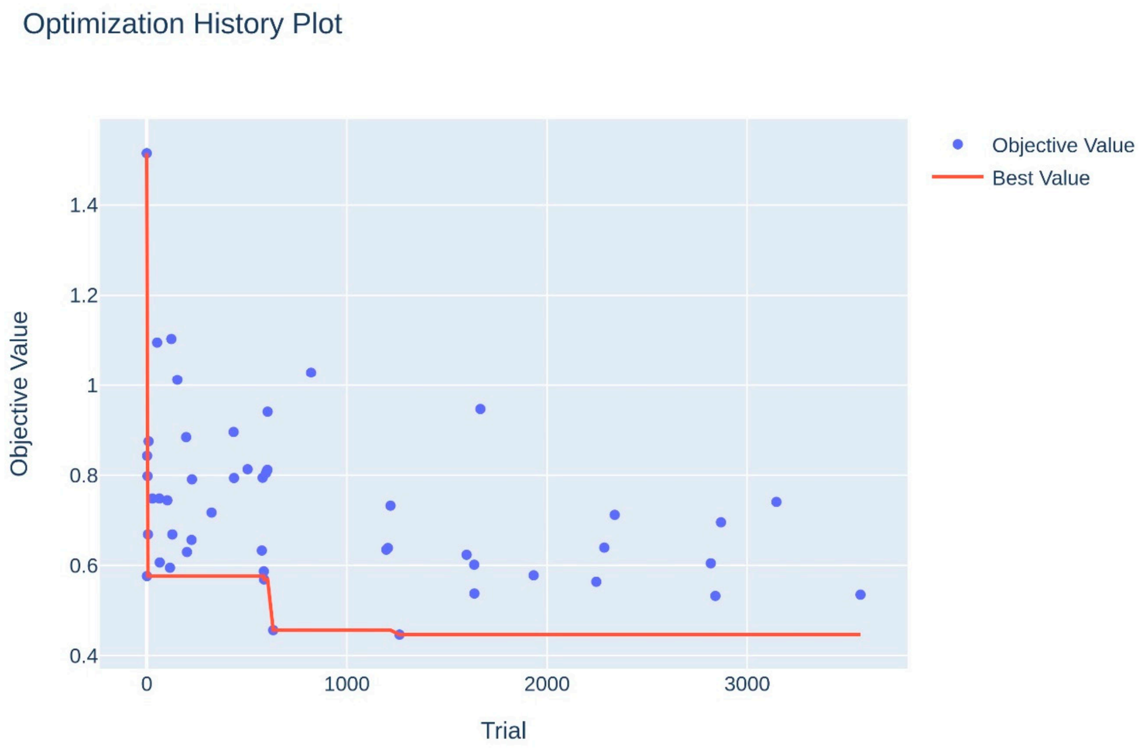
Optimization History Plot
Objective Value
Best Value
1.4
1.2
1
0.8
0.6
0.4
Objective Value
0
1000
2000
3000
Trial
